# Predicting Risk of Post-Operative Morbidity and Mortality following Gynaecological Oncology Surgery (PROMEGO): A Global Gynaecological Oncology Surgical Outcomes Collaborative Led Study

**DOI:** 10.3390/cancers16112021

**Published:** 2024-05-26

**Authors:** Faiza Gaba, Sara Mahvash Mohammadi, Mikhail I. Krivonosov, Oleg Blyuss, on behalf of the GO SOAR Collaborators

**Affiliations:** 1 Department of Gynaecological Oncology, The Royal Marsden Hospital, London SW3 6JJ, UK; 2 Institute of Applied Health Sciences, University of Aberdeen, Aberdeen AB24 3FX, UK; 3 Centre for Cancer Screening, Prevention and Early Detection, Wolfson Institute of Population Health, Queen Mary University of London, London EC1M 6BQ, UK; 4 Research Center for Trusted Artificial Intelligence, Ivannikov Institute for System Programming of the Russian Academy of Sciences, Moscow 109004, Russia; 5 Institute of Biogerontology, Lobachevsky State University, Nizhny Novgorod 603105, Russia; 6 Department of Pediatrics and Pediatric Infectious Diseases, Institute of Child’s Health, Sechenov University, Moscow 119991, Russia

**Keywords:** surgical risk calculator, machine learning, surgical morbidity, surgical mortality

## Abstract

**Simple Summary:**

Accurate pre-operative surgical risk predictions form the foundation of pre-operative counseling and informed consent. There are currently no validated risk calculators that are able to accurately predict post-operative complications for women undergoing gynecological cancer surgery in both high- and low-middle-income healthcare settings. Using the dataset from the international GO SOAR database, we present a novel artificial intelligence surgical risk calculator capable of accurately predicting the risk of complications associated with gynecological cancer surgery. The GO SOAR surgical risk calculator uses readily available pre-operative data available across all-income healthcare settings, ensuring benefits to women globally.

**Abstract:**

The medical complexity of surgical patients is increasing, and surgical risk calculators are crucial in providing high-value, patient-centered surgical care. However, pre-existing models are not validated to accurately predict risk for major gynecological oncology surgeries, and many are not generalizable to low- and middle-income country settings (LMICs). The international GO SOAR database dataset was used to develop a novel predictive surgical risk calculator for post-operative morbidity and mortality following gynecological surgery. Fifteen candidate features readily available pre-operatively across both high-income countries (HICs) and LMICs were selected. Predictive modeling analyses using machine learning methods and linear regression were performed. The area-under-the-receiver-operating characteristic curve (AUROC) was calculated to assess overall discriminatory performance. Neural networks (AUROC 0.94) significantly outperformed other models (*p* < 0.001) for evaluating the accuracy of prediction across three groups, i.e., minor morbidity (Clavien–Dindo I-II), major morbidity (Clavien–Dindo III-V), and no morbidity. Logistic-regression modeling outperformed the clinically established SORT model in predicting mortality (AUROC 0.66 versus 0.61, *p* < 0.001). The GO SOAR surgical risk prediction model is the first that is validated for use in patients undergoing gynecological surgery. Accurate surgical risk predictions are vital within the context of major cytoreduction surgery, where surgery and its associated complications can diminish quality-of-life and affect long-term cancer survival. A model that requires readily available pre-operative data, irrespective of resource setting, is crucial to reducing global surgical disparities.

## 1. Introduction

There is considerable uncertainty in health care, and risk prediction plays a fundamental role in a surgeon’s ability to drive clinical decisions, counsel patients, and evaluate outcomes. Studies have shown that clinicians are imperfect when predicting medical and surgical risk and often rely on their experience and subjective global assessment of patient fitness for surgery [1,2,3,4]. Surgical risk calculators are a set of tools with the potential to mitigate the highly variable perception of patient risk [5,6,7,8].

Quality and safety remain essential to the practice of all surgery, and implicit in this process is the accurate risk assessment of planned surgical procedures using surgical risk calculators [9,10,11]. To engage in a meaningful process of informed consent and mitigate anticipated surgical risks, patient, disease, and surgical factors must be considered in a robust risk assessment. Application of this information using surgical risk calculators can clarify the risk-to-benefit profile of surgery, particularly within the context of major cytoreduction surgery, which can often involve multiple visceral organ resections impacting quality of life [12,13,14]. Risk calculators are important instruments for shared decision-making between patients and doctors [13,15,16]. However, pre-existing surgical risk calculators are limited in their ability to accurately predict risk for major gynecological oncology surgeries and are not validated for use in such a population. In addition, the applicability of pre-existing calculators is limited in low-income resource settings, as restricted resources preclude the widespread use of biochemical and radiological tests, and even stable internet access limits the utility of some technologies for risk prediction. In order to inform consent and shared decision-making, a robust, globally applicable surgical risk prediction model is needed to predict individualized morbidity and mortality risk for patients undergoing gynecological oncology surgery. The aim of this study is to develop a novel machine learning-based surgical risk calculator to accurately predict thirty-day postoperative morbidity and mortality risk in women undergoing gynecological oncology surgery in high- and low-middle-income country settings.

## 2. Materials and Methods

### 2.1. Source Data and Participants

An international, multicenter, prospective cohort study (GO SOAR1, NCT04579861) included consecutive patients undergoing surgery for ovary, uterus, cervix, vulva, and vaginal cancers over a thirty-day period in seventy-three hospitals across twenty-seven countries in low-middle-income (LMIC) and high-income (HIC) settings. Patients undergoing elective and emergency surgeries were included between January 2021 and November 2022. Inclusion criteria were women aged ≥ 18 years undergoing curative or palliative surgery for primary or recurrent gynecological malignancies. The surgical modalities included were open, minimal access (laparoscopic and robotic), and vaginal. Elective and emergency cases were included. Patients were excluded if their primary pathology was not a gynecological malignancy, benign, or borderline disease, and if they had undergone a diagnostic procedure. Investigators were required to monitor patients for a minimum of thirty days post-operatively to identify complications. A full study methodology has been published previously [17,18].

The data collected on the prospective GO SOAR database as part of the GO SOAR1 study were used to conduct predictive modeling analyses in two separate settings, i.e., one to discriminate between minor (Clavien–Dindo I–II) and major morbidity (Clavien–Dindo III–V) from a group without morbidity (analysis 1), and the second to discriminate between individuals who died and those who survived (analysis 2) thirty days from surgery. The study has been approved and registered with the School Ethics Review Board for the School of Medicine, Medical Sciences at the University of Aberdeen, UK (SERB/2021/10/2194).

### 2.2. Candidate Predictor Variables

We planned to include variables that are readily available globally, even in resource-limited environments, without the need for additional tests. To enable this model to inform pre-operative decision-making, we only selected variables that are systematically available before surgery. To achieve this, sixteen candidate predictors were selected a priori to be included and processed. These were selected from three domains, i.e., patient, disease, and surgical predictors. Patient predictors included the following: age (linear); ethnicity (white versus non-white); body mass index (kg/m^2^, linear); hemoglobin (g/dL, linear); white cell count (10^9^/L, linear); albumin (g/L, linear); American Society of Anesthesiologists (ASA) grade (1–2 versus 3–5); and Eastern Cooperative Oncology Group (ECOG) performance status (0–2 versus 3–4). Disease predictors included the following: primary cancer (ovary, uterine, cervical, vulva/vagina); radiological FIGO stage (stage I–II versus stage III–IV); and neoadjuvant chemotherapy (yes versus no). Surgical predictors included the following: history of previous abdominal surgery (minimal access (laparoscopy/robotic) versus laparotomy); mechanical bowel preparation (yes versus no); intra-operative antibiotics (yes versus no); surgical modality (minimal access versus laparotomy); and surgical complexity score (estimated pre-operatively based on radiological imaging, low = ≤8, moderate = 9–16, high = ≥16). The surgical complexity score was divided into five separate groups, i.e., pelvic surgery, bowel surgery, urological surgery, upper abdominal surgery, and lymphadenectomy. Each of these five groups was further subdivided into specific surgical procedures and allocated a complexity score based on expert consensus (Supplementary Table S1).

### 2.3. Missing Data

From the sixteen candidate predictors, a predictor was excluded if ≥20% of values were missing. Analyses were performed using both complete cases and an imputed dataset. Missing data were handled using the multivariate imputation by chained equations (MICE) method, generating five different datasets [19]. This approach allowed for more robust analysis by retaining all available information while addressing missing data.

### 2.4. Model Building and Validation

For analysis 1, we employed the following machine learning methods: support vector machines, random forests, gradient boosting, and feedforward neural networks. These methods were chosen based on their established performance in similar predictive tasks. For each method, leave-one-out cross-validation (LOOCV) was utilized to evaluate the accuracy of prediction across the three classes, i.e., minor morbidity, major morbidity, and no morbidity. For every patient, each of the methods was trained using all the data but the present patient, and then the resulting model was used to make a prediction for the index patient. To account for the fact that the data were imbalanced, the synthetic minority oversampling technique (SMOTE) was used at each LOOCV step. Because the outcome was not binary but rather categorical, with three classes, no feature selection was employed. Additionally, the multiclass area under the receiver operating characteristic curve (AUROC) was calculated to assess the overall discriminatory performance [6]. The statistical significance of differences in accuracies among methods within each class was evaluated using McNemar’s test.

For analysis 2, we aimed to assess whether logistic regression, a straightforward binary classification approach, could outperform the SORT (surgical outcome risk tool) calculator, which is an established risk prediction calculator for predicting postoperative mortality in clinical practice [20]. Given the limited sample size, logistic regression was chosen as it offers simplicity and interpretability. We employed Monte Carlo cross-validation with 1000 iterations, splitting the data into training and testing sets in a 50:50 ratio at each step. Logistic regression models were trained using the training set and then evaluated on the test set. At each training step, feature selection was employed based on Akaike’s information criterion (AIC) to identify a subset of the most predictive features, which were then used for the test set. Performance was compared against the SORT calculator in terms of AUROC and sensitivity at a clinically sensible specificity threshold of 90%. The Wilcoxon rank sum test was used to assess the significance of differences in AUROC measures and sensitivities between logistic regression and SORT. All statistical tests were two-sided, and a *p* value < 0.05 was considered statistically significant.

The analyses were performed using Python version 3.8 and R version 3.5.1. This prediction model is reported in alignment with TRIPOD [21] and PROBAST [22] guidelines.

## 3. Results

Analysis 1 included 1310 patients with no morbidity, 374 patients with minor morbidity, and 127 patients with major morbidity. Table 1 summarizes the spread of candidate predictors between the three groups. Of the sixteen a priori candidate predictors selected, fifteen were used in analysis 1. Albumin was excluded as it was missing in 71% of cases in the entire dataset.

All continuous variables are described through the median and the 25th and 75th percentiles. Categorical variables are shown with percentages, and *p* values were calculated depending on the type of variable with Kruskal–Wallis for continuous and the chi-squared or Fisher test for categorical.

**Figure 1:**
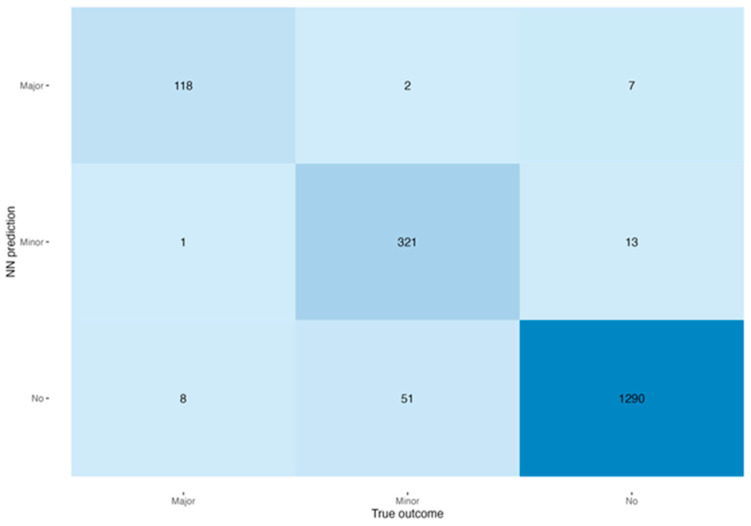
Heatmap of the confusion matrix of the performance of the neural networks. Shaded areas represent accuracy of respective true outcome.

Table 2 shows accuracies in each of the three groups of patients for every method as well as multiclass AUROC values. The neural network approach significantly outperformed other methods in each of the groups (*p* ≤ 0.001), with a multiclass AUROC of 0.94. The neural network model had accuracies of 98.5% (1290/1310), 85.8% (321/374), and 92.9% (118/127) for predicting no morbidity, minor morbidity, and major morbidity, respectively. Figure 1 shows the confusion matrix for the performance of the neural networks as the best-performing approach.

**Figure 2:**
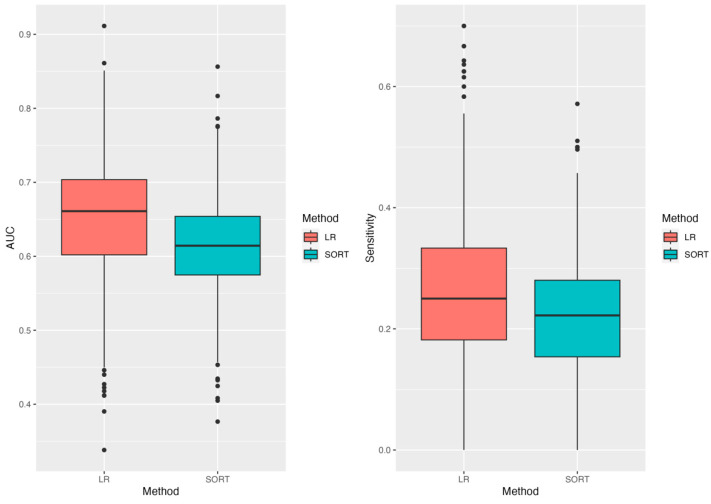
Distribution of the AUROC and sensitivities values across 1000 splits for the logistic regression and SORT.

Analysis 2 included 24 patients with postoperative deaths and 1787 without. Table 3 summarizes the spread of candidate predictors between the three groups. Albumin was once again excluded. Table 4 shows AUROC and sensitivity values at a specificity of 0.9 for the logistic regression and SORT based on the Monte Carlo cross-validation approach. Logistic regression significantly outperformed SORT for both parameters (*p* ≤ 0.001). Figure 2 shows boxplots reflecting the AUROCs and the sensitivities at a specificity of 90% for the logistic regression and for SORT. Logistic regression and SORT had an AUROC of 0.66 and 0.61, respectively, and sensitivity at a specificity of 0.9 of 0.25 and 0.22, respectively. Supplementary Table S2 summarizes the final clinical features for each iteration of Monte Carlo cross-validation that has been used to develop the linear regression model. The frequency of being incorporated into the final model for each clinical feature was calculated. Age, surgery involving gastrointestinal, urological, vascular, or thoracic procedures, FIGO stage, ethnicity, and performance status were clinical features with the highest frequencies.

All continuous variables are described through the median and the 25th and 75th percentiles. Categorical variables are shown with percentages, and *p* values were calculated depending on the type of variable with the Wilcoxon rank-sum test for continuous and the chi-squared or Fisher test for categorical.

## 4. Discussion

In this study, we present the first internally validated machine learning risk prediction model that is capable of accurately predicting thirty-day postoperative morbidity and mortality for women undergoing major gynecological oncology surgery. The GO SOAR surgical risk calculator is globally applicable and consists of variables that are readily available across all resource settings. The model is derived and validated in a global dataset (seventy-three hospitals, across twenty-seven countries).

Within peri-operative gynecological oncology surgical practice, widely used surgical risk calculators include the American College of Surgeons National Surgical Quality Improvement Program (ACS NSQIP) and SORT. The ACS NSQIP surgical risk calculator is designed to predict the risk of any complication, any serious complication (defined as death, cardiac arrest, myocardial infarction, pneumonia, progressive renal insufficiency, acute renal failure, pulmonary embolus, deep venous thrombosis, return to theatre, deep incisional surgical space infection (SSI), organ space SSI, systemic sepsis, unplanned intubation, urinary tract infection (UTI), wound disruption), seven individual postoperative serious complications, readmission, length of stay, and discharge to post-acute care. The calculator was originally developed using a regression model to determine the strength of the association between pre-operative variables and postoperative outcomes using data from 1.4 million patients at 393 NSQIP hospitals. The variables within the calculator were weighted based on the regression coefficient [23,24]. Data from all surgical specialties except trauma and transplant were included in the development of the calculator. However, patients undergoing gynecological surgery consist of only 5.3% of the original cohort, and only 1.1% of the population was used to develop the discharge-to-post-acute care prediction tool [23,24]. Due to the widespread use of the ACS NSQIP calculator within gynecological oncology, there have been numerous retrospective studies that have attempted to validate it within a gynecological oncology cohort. All of these studies, without exception, have found the predictive ability of the ACS NSQIP calculator in gynecological oncology patients to be inferior compared to its performance within a colorectal surgical cohort, which served as the original validation dataset for the calculator [23,25,26,27].

Attempts to validate other multi-specialty surgical risk calculators, such as the National Surgical Quality Improvement Program Universal Surgical Risk Calculator (derived from the ACS NSQIP dataset), within gynecological oncology have also shown poor performance and inaccurate risk predictions [23,28].

A major limitation of pre-existing calculators is that while they consider patient factors, there is little to no consideration of disease and surgical complexity. In part due to the usage of these risk calculators across multiple surgical specialties and across both oncological and benign settings, the incorporation of a cross-specialty surgical complexity score would be challenging and could not account for all the different specialty-specific surgeries. To overcome this limitation and improve the accuracy of risk prediction, the development and clinical validation of specialty-specific risk prediction calculators are crucial.

The generalizability of pre-existing surgical risk prediction calculators is limited within resource-poor LMIC settings for multiple reasons [29]. Firstly, calculators developed require additional tests that are not routinely performed pre-operatively. For example, the physiological and operative severity score for the enumeration of mortality and morbidity (POSSUM) surgical risk calculator [30] requires a blood urea nitrogen (BUN) entry. The pre-operative laboratory results, collected as part of our international prospective GO SOAR database, indicate that this is not a widely performed test in LMIC healthcare facilities. Secondly, the datasets used to validate risk calculators such as ACS NSQIP, SORT, and POSSUM are from HIC settings and are not representative of LMIC populations. Thirdly, there is a lack of robust validation of pre-existing surgical risk calculators within LMIC settings.

Strengths of our study include the development and internal validation of the first gynecological oncology-specific surgical risk prediction calculator. In addition, because the GO SOAR surgical risk prediction calculator has been derived from a large perspective dataset incorporating both HIC and LMIC populations, it may be used across all income and resource settings as the data points required are widely available pre-operatively, irrespective of resources and infrastructure. This will also enable individuals identified as being at increased risk of postoperative surgical morbidity to access pre-operative prehabilitation, thereby reducing disparities in surgical morbidity between HIC and LMIC settings.

Limitations include the small number of deaths in the dataset that was used to validate our model. The GO SOAR database is continuing to capture HIC and LMIC data, and future collected data will be used to refine our model and improve mortality prediction. In addition, future work is planned to externally validate the GO SOAR surgical risk calculator to identify patients at low, moderate, or high risk of post-operative morbidity and mortality. It was not possible to compare the performance of our model’s morbidity prediction to pre-existing models such as ACS NSQIP. This was due to the inability to accurately match the extent of gynecological oncology surgery (particularly ovarian cancer cytoreduction surgeries) to the very limited gynecological surgical options available as part of the ACS NSQIP calculator. Mismatched selections are a key reason for the poor performance of the ACS NSQIP calculator in the multiple retrospective studies that have attempted to validate the calculator within a gynecological oncology population.

Accurate pre-operative surgical risk predictions form the cornerstone of pre-operative counseling and informed consent. Particularly within the context of major cytoreduction surgery, where surgery and its associated complications can diminish quality of life and affect long-term cancer survivorship. It is important that women with gynecological malignancies globally are able to make informed decisions balancing cancer survival and quality of life following major surgery.

## 5. Conclusions

The medical complexity of surgical patients is increasing, and surgical risk calculators are a valuable tool in providing high-value, patient-centered surgical care. The GO SOAR surgical risk calculator outperforms the SORT surgical risk prediction calculator that is widely in use in gynecological oncology clinical practice. Accurate surgical risk calculators that can be used in both HIC and LMIC settings are important to reduce international disparities in surgical care.

## Figures and Tables

**Table 1:** Candidate predictors of patients included in analysis 1.

Candidate Predictor	Subgroups	No Morbidity N = 1310	Minor Morbidity N = 374	Major Morbidity N = 127	*p*-Value
Age	Median (IQR)	61 ((51–69)	60 (52–69)	62 (53–71)	0.331
Ethnicity	Non-white	442 (33.7%)	150 (40.1%)	46 (36.2%)	0.073
	White	868 (66.3%)	224 (59.9%)	81 (63.8%)	
BMI	Median (IQR)	27.2 (23.5–32)	27.3 (23.2–32)	26 (21.8–29.2)	<0.001
ASA grade	3, 4	272 (20.8%)	109 (29.1%)	32 (25.2%)	0.002
	1, 2	1038 (79.2%)	265 (70.9%)	95 (74.8%)	
ECOG status	4, 5	19 (1.5%)	4 (1.1%)	6 (4.7%)	0.012
	1, 2, 3	1291 (98.5%)	370 (98.9%)	121 (95.3%)	
Previous laparotomy	No	870 (66.4%)	222 (59.4%)	68 (53.5%)	0.002
	Yes	440 (33.6%)	152 (40.6%)	59 (46.5%)	
Previous laparoscopy	No	1049 (80.1%)	265 (70.9%)	91 (71.7%)	<0.001
	Yes	261 (19.9%)	109 (29.1%)	36 (28.3%)	
Pre-operative haemoglobin	Median (IQR)	14.3 (12.4–127)	14.2 (12–123.8)	13.2 (11.7–108)	0.002
Pre-operative white cell count	Median (IQR)	7.1 (5.7–8.9)	7 (5.5–9.4)	7.4 (6–9.5)	0.318
Neoadjuvant chemotherapy	No	437 (33.4%)	138 (36.9%)	50 (39.4%)	0.219
	Yes	873 (66.6%)	236 (63.1%)	77 (60.6%)	
Surgical modality	Laparotomy	720 (55%)	279 (74.6%)	91 (71.7%)	<0.001
	Laparoscopic or robotic	590 (45%)	95 (25.4%)	36 (28.3%)	
Mechanical bowel preparation	No	730 (55.7%)	152 (40.6%)	63 (49.6%)	<0.001
	Yes	580 (44.3%)	222 (59.4%)	64 (50.4%)	
Intra-operative antibiotics	No	205 (15.6%)	37 (9.9%)	14 (11%)	0.01
	Yes	1105 (84.4%)	337 (90.1%)	113 (89%)	
FIGO stage	III, IV	436 (33.3%)	200 (53.5%)	74 (58.3%)	<0.001
	I, II	874 (66.7%)	174 (46.5%)	53 (41.7%)	
Primary cancer	Cervix	117 (8.9%)	38 (10.2%)	10 (7.9%)	<0.001
	Endometrium	597 (45.6%)	120 (32.1%)	38 (29.9%)	
	Ovary	503 (38.4%)	179 (47.9%)	64 (50.4%)	
	Vagina	8 (0.6%)	6 (1.6%)	0 (0%)	
	Vulva	85 (6.5%)	31 (8.3%)	15 (11.8%)	
Surgical complexity score	Low	907 (69.2%)	193 (51.6%)	53 (41.7%)	<0.001
	Moderate	341 (26%)	119 (31.8%)	43 (33.9%)	
	High	62 (4.7%)	62 (16.6%)	31 (24.4%)	

**Table 2:** Performance based on leave-one-out cross-validation.

Machine Learning Methodology	Accuracy No Morbidity	Accuracy Minor Morbidity	Accuracy Major Morbidity	Multiclass AUROC
SVM	92.3%	17.4%	9.4%	0.565
RF	88.5%	24.9%	11%	0.581
GB	87.3%	25.7%	15.7%	0.581
NN	98.5%	85.8%	92.9%	0.941

SVM—support vector machines; RF—random forest; GB—gradient boosting; NN—neural networks.

**Table 3:** Candidate predictors of patients included in analysis 2.

Candidate Predictors	Subgroups	Alive N = 1787	Dead N = 24	*p* Value
Age	Median (IQR)	61 (51–69)	68.5 (59.3–76)	<0.001
Ethnicity	Non-white	624 (34.9%)	14 (58.3%)	0.03
	White	1163 (65.1%)	10 (41.7%)	
BMI	Median (IQR)	27 (23.1–32)	28.2 (26.9–32.9)	0.182
ASA grade	3, 4	402 (22.5%)	11 (45.8%)	0.014
	1, 2	1385 (77.5%)	13 (54.2%)	
ECOG status	4, 5	26 (1.5%)	3 (12.5%)	<0.001
	1, 2, 3	1761 (98.5%)	21 (87.5%)	
Previous laparotomy	No	1144 (64%)	16 (66.7%)	0.957
	Yes	643 (36%)	8 (33.3%)	
Previous laparoscopy	No	1384 (77.4%)	21 (87.5%)	0.354
	Yes	403 (22.6%)	3 (12.5%)	
Pre-operative haemoglobin	Median (IQR)	14.2 (12.3–126)	12.8 (10.6–104.3)	0.025
Pre-operative white cell count	Median (IQR)	7.1 (5.7–187.4)	7.6 (6–11.9)	0.177
Neoadjuvant chemotherapy	No	613 (34.3%)	12 (50%)	0.164
	Yes	1174 (65.7%)	12 (50%)	
Surgical modality	Laparotomy	1072 (60%)	18 (75%)	0.2
	Laparoscopic or robotic	715 (40%)	6 (25%)	
Mechanical bowel preparation	No	933 (52.2%)	12 (50%)	0.992
	Yes	854 (47.8%)	12 (50%)	
Intra-operative antibiotics	No	252 (14.1%)	4 (16.7%)	0.766
	Yes	1535 (85.9%)	20 (83.3%)	
FIGO stage	III, IV	693 (38.8%)	17 (70.8%)	0.003
	I, II	1094 (61.2%)	7 (29.2%)	
Primary cancer	Cervix	165 (9.2%)	0 (0%)	0.254
	Endometrium	741 (41.5%)	14 (58.3%)	
	Ovary	736 (41.2%)	10 (41.7%)	
	Vagina	14 (0.8%)	0 (0%)	
	Vulva	131 (7.3%)	0 (0%)	
Surgical complexity score	Low	152 (8.5%)	3 (12.5%)	0.135
	Moderate	1142 (63.9%)	11 (45.8%)	
	High	493 (27.6%)	10 (41.7%)	

**Table 4:** Comparison of logistic regression with AIC-based feature selection with SORT using Monte Carlo cross-validation.

Model	AUROC	Sensitivity at a Specificity = 0.9
Logistic regression	0.661 (0.602–0.704)	0.25 (0.182–0.333)
SORT	0.614 (0.575–0.654)	0.222 (0.154–0.28)

Note: 1000 splits into 50%:50%.

## Data Availability

Relevant anonymized data can be obtained upon reasonable request from the corresponding author.

## Supplementary Materials

The following supporting information can be downloaded at: https://www.mdpi.com/article/10.3390/cancers16112021/s1, Table S1: Surgical complexity score. Table S2: Frequencies of candidate predictors for each iteration of Monte-Carlo cross-validation that has been used to develop the final linear regression model.
