# Supplementary material for: Predicting Risk of Post-Operative Morbidity and Mortality following Gynaecological Oncology Surgery (PROMEGO): A Global Gynaecological Oncology Surgical Outcomes Collaborative Led Study
Source: Cancers (Basel). 2024 May 26;16(11):2021. doi: 10.3390/cancers16112021 (PMC11170986; doi:10.3390/cancers16112021)
Supplement: Supplementary file 1 [file cancers-16-02021-s001.zip › cancers-2999215-supplementary tables.pdf]

Table S1: Surgical complexity score

Pelvic surgery	Score
Uterus	1
Cervix	1
Fallopian tube(s)	1
Ovary(s)	1
Upper vagina	2
Parametrium	2
Pelvic peritoneum	2
Total vagina	3
Obturator internus	3
Anus	3
Vulva	3
Sciatic nerve	4
Psoas major	4
Levator ani	4
**Bowel surgery**	
Omentum	2
Abdominal peritoneum	2
Appendix	2
Mucous fistula	2
Large bowel stoma	2
Small bowel stoma	2
Rectum	3
Sigmoid	3
Ascending colon	3
Descending colon	3
Transverse colon	3
Duodenum	3
Jejunum	3
Ileum	3
Large bowel anastomosis	4
Small bowel anastomosis	4
**Urological surgery**	
Nephrostomy	2
Bladder	3
Ureter	3
Kidney	3
Urostomy/ileal conduit	3
Ureteric reimplantation	4
Trans uretero-ureteric anastomosis	4
**Upper abdominal surgery**	
Spleen	3
Liver capsule	3
Gall bladder	3
Diaphragm resection/stripping	3
Liver parenchyma	4
Stomach	4
Pancreas	4

**Lymphadenectomy**	
Pelvic	3
Para-aortic	3
Inguinal	3

Table S2: Frequencies of candidate predictors for each iteration of Monte-Carlo cross-validation that has been used to develop the final linear regression model

Candidate predictor	Frequency (%) of being incorporated into the final linear regression model
Age	73.4
*Surgery involving gastrointestinal/urological/vascular/thoracic procedures	73.3
FIGO stage	61.6
Ethnicity	55.4
ECOG status	53.9
Neoadjuvant chemotherapy	45
BMI	30.8
Previous minimal access surgery (laparoscopy/robotic)	26.5
Tumour origin	26.4
ASA grade	24.3
Intra-operative antibiotics	22.4
Mechanical bowel preparation	20.7
Surgical modality	18.4
Surgical complexity score	18.1
*Urgency of surgery (elective/emergency)	13.5
Previous laparotomy	13.2
Preoperative haemoglobin	7.8
Preoperative white cell count	3.7

*SORT calculator specific variables
